# Central Sympathetic Activation and Arrhythmogenesis during Acute Myocardial Infarction: Modulating Effects of Endothelin-B Receptors

**DOI:** 10.3389/fcvm.2015.00006

**Published:** 2015-02-23

**Authors:** Theofilos M. Kolettis, Marianthi Kontonika, Eleonora Barka, Evangelos P. Daskalopoulos, Giannis G. Baltogiannis, Christos Tourmousoglou, Apostolos Papalois, Zenon S. Kyriakides

**Affiliations:** ^1^Cardiovascular Research Institute, Ioannina and Athens, Greece; ^2^Experimental Research Center ELPEN, Athens, Greece

**Keywords:** acute myocardial infarction, sympathetic activation, ventricular arrhythmias, endothelin receptors

## Abstract

Sympathetic activation during acute myocardial infarction (MI) is an important arrhythmogenic mechanism, but the role of central autonomic inputs and their modulating factors remain unclear. Using the *in vivo* rat-model, we examined the effects of clonidine, a centrally acting sympatholytic agent, in the presence or absence of myocardial endothelin-B (ETB) receptors. We studied wild-type (*n* = 20) and ETB-deficient rats (*n* = 20) after permanent coronary ligation, with or without pretreatment with clonidine. Cardiac rhythm was continuously recorded for 24 h by implantable telemetry devices, coupled by the assessment of autonomic and heart failure indices. Sympathetic activation and arrhythmogenesis were more prominent in ETB-deficient rats during the early phase post-ligation. Clonidine improved these outcomes throughout the observation period in ETB-deficient rats, but only during the delayed phase in wild-type rats. However, this benefit was counterbalanced by atrioventricular conduction abnormalities and by higher incidence of heart failure, the latter particularly evident in ETB-deficient rats. Myocardial ETB-receptors attenuate the arrhythmogenic effects of central sympathetic activation during acute MI. ETB-receptor deficiency potentiates the sympatholytic effects of clonidine and aggravates heart failure. The interaction between endothelin and sympathetic responses during myocardial ischemia/infarction and its impact on arrhythmogenesis and left ventricular dysfunction merits further investigation.

## Introduction

Sudden cardiac death (SCD) accounts for 13% of total mortality from natural causes in the general population and has emerged as an important health-related problem. In most cases, SCD is caused by ventricular tachyarrhythmias, i.e., ventricular tachycardia (VT) and ventricular fibrillation (VF), triggered by acute myocardial infarction (MI) (1). Driven by its high-societal impact, in-depth understanding of the pathophysiology of ischemia-induced VT/VF has attracted multifaceted research efforts, with a view toward decreasing SCD rates.

The role of sympathetic activation in the pathogenesis of VT/VF during acute MI has been known for decades (2); myocardial ischemia leads to local norepinephrine release from sympathetic nerve terminals, primarily via non-exocytotic mechanisms, following an immediate exocytotic phase (3). The elicited positive inotropic response serves the maintenance of cardiac output, but local catecholamine surge alters ventricular electrophysiological properties and creates an arrhythmogenic milieu (4). These mechanisms may predominate during the early period post-MI, corresponding clinically to the critical pre-hospital stage, during which VT/VF carries a dismal prognosis (1).

Recently, accumulated evidence indicates endothelin-1 (ET-1), a ubiquitous 21-aminoacid peptide, as an important moderator of sympathetic activation (5) and arrhythmogenesis (6) during acute-MI. Both (ETA and ETB) ET-receptors, located in sympathetic nerve varicosities in the ventricular myocardium (7), exert significant, albeit opposing effects; ETA-receptors inhibit norepinephrine re-uptake, whereas ETB-receptors attenuate exocytotic norepinephrine release (8). These actions gain pathophysiological significance in the MI-setting, during which ETA-receptor stimulation enhances norepinephrine release, but the role of ETB-receptors remains unclear (9). Experimental studies have shown increased sympathetic activation and arrhythmogenesis in the absence of functional ETB-receptors in the ischemic ventricular myocardium (7, 10); however, most data originate from *ex vivo*, isolated (hence, denervated) beating hearts, thereby hindering the deduction of firm conclusions on the role of ETB-receptors, in the presence of intact autonomic innervation (11).

In addition to myocardial norepinephrine release from intrinsic nerve terminals, central sympathetic activation occurs after ischemia, mediated by locally produced metabolites that stimulate afferent myocardial nerve fibers; in turn, efferent autonomic discharges from the brain stem modulate left ventricular (LV) function and electrophysiology (12). Earlier studies in anesthetized dogs demonstrated increased cardiac sympathetic nerve activity during MI that was prevented by clonidine, a centrally acting α_2_-adrenergic-receptor agonist (13). Efferent inputs to the heart are arrhythmogenic, as shown by continuous recordings of the left stellate ganglion in conscious dogs, revealing the precedence of VT/VF by enhanced sympathetic nerve activity (14), and by effective clinical management of intractable VT/VF by surgical or thoracoscopic denervation (15). Despite this knowledge, the relative impact of central sympathetic activation on VT/VF along with the course of acute-MI, and the potential modulating effects of ETB-receptors in the ventricular myocardium are not well defined.

Here, we examined arrhythmogenesis, as well as indices of sympathetic activation and LV failure, in the *in vivo* acute-MI rat-model; we compared these outcomes in groups with or without pretreatment with clonidine, in the presence or absence of myocardial ETB-receptors.

## Materials and Methods

### Animal study population and ethics

The animal study population consisted of 40 rats, 20–24 weeks of age, weighing 225–300 g. The animals were housed in plexiglas-cages in groups of two, with free access to standard rodent pellet-diet and water. The laboratory conditions were kept optimal, in terms of temperature (20–22°C), humidity (~70%), and light/dark cycles (12/12 h). The study protocol adheres to the guiding principles of the declaration of Helsinki (on animal research) and to European legislation (*European Union directive for the protection of animals used for scientific purposes* 609/1986, revised in 2010/63/EU); all procedures were approved by the institutional ethics’ committee and by the regulatory state authorities.

The role of ETB-receptors in the ventricular myocardium was examined by a “subtraction model,” using a previously characterized (16) Wistar–Imamichi rat strain (*n* = 20), kindly provided by Professor M. Yanagisawa (Southwestern Medical Center, Dallas, TX, USA and University of Tsukuba, Tsukuba, Japan). These animals carry a (naturally occurring) deletion in the gene encoding for the ETB-receptor and die prematurely of intestinal obstruction. This phenotype is rescued by directed ETB transgene-expression, leading to normal development of enteric nervous system and brain function, and provides a valuable tool in the study of ETB-receptors in the cardiovascular system (17).

### Study protocol

Cardiac rhythm was recorded continuously for 24 h post-MI, with the use of miniature electrocardiography (ECG) telemetry transmitters; these devices permit long-term assessment in conscious animals, without the confounding effects of anesthesia (18). The survival duration was accurately determined from the stored ECG-recordings; mortality was further classified as tachyarrhythmic (i.e., ventricular asystole, immediately preceded by VT/VF) or bradyarrhythmic [i.e., gradual increase in sinus heart rate (HR), followed by an abrupt onset of complete atrioventricular block and asystole], the latter indicative of death due to heart failure (18, 19).

### Transmitter implantation

The telemetry transmitters (Dataquest, Data Sciences International, *DSI*, Transoma Medical, Arden Hills, MN, USA) were implanted in the abdominal cavity, as described previously (20); both leads were tunneled under the skin and secured at the right axillary and left inguinal areas, respectively. The animals were housed in individual cages, placed on a receiver that continuously captured the ECG-signal, processed by a software program (A.R.T. 2.2, *DSI*).

### Clonidine administration

Central sympathetic activation was examined with the use of clonidine, a commonly applied pharmacological model; after an initial peripheral α_1_-stimulation, the central action of clonidine prevails, resulting in inhibition of sympathetic preganglionic neurons and decreased sympathetic drive (21). As previously (22), clonidine was given intraperitoneally (0.5 mg/kg), 1 h prior to the experiments.

### Induction of myocardial infarction

After tracheal intubation, the rats were mechanically ventilated (rodent apparatus model 7025, Ugo Basile, Comerio, Italy), and anesthesia was maintained with a mixture of oxygen and 2.5% sevoflurane. MI was generated by permanent ligation at the middle segment of the left coronary artery, guided by the anatomic landmarks provided by the pulmonary cone, the left atrium, and the ventricular apex (23). MI was validated by inspection of a pale, akinetic area, and by ST-segment elevation in a six-lead ECG (QRS-Card digital PC-ECG, Pulse Biomedical Inc., *PBI*, Norristown, PA, USA, amplified by *Cardiology Suite*, version 4.05, *PBI*). The incision was closed in three layers and pneumothorax was evacuated. Spontaneous respiration and consciousness resumed within ~2 min after discontinuation of anesthesia.

### Arrhythmia analysis

The analysis of ECG-recordings adhered to the guidelines provided by the (recently updated) Lambeth conventions for determination of experimental arrhythmias (24). The tracings were manually scrolled and the number of tachy- and bradyarrhythmic events were recorded; the count of premature ventricular contractions, couplets, or triplets was omitted, based on their uncertain significance. As distinction between VT (four or more consecutive premature ventricular contractions) and VF (indistinguishable QRS deflections) may be occasionally difficult (19, 20), we report them collectively as VT/VF; the duration of each episode was measured, aided by the time-scale provided by the software. Similarly, we recorded transient atrioventricular conduction abnormalities, not associated with bradyarrhythmic death. To account for differences in mortality, VT/VF duration during the entire observation period was normalized for survival (18).

### Arrhythmia time-intervals

The incidence of VT/VF is reported separately for phase I (i.e., during the first hour post-ligation, corresponding to ischemia and the onset of myocardial necrosis), and phase II (i.e., from the 2nd until the 24th hour post-ligation, corresponding to evolving MI, until the completion of necrosis). Such separation presents the inherent limitation of producing temporal dissimilarities in the characteristics within an animal-population in cases of excessive early-phase mortality, but it is useful for its translational value in the clinical setting, and also provides information on the underlying arrhythmogenic mechanisms (25, 26).

### ECG-indices of sympathetic activation

Mean values of HR, calculated from sinus beats, are given separately for phases I and II. Furthermore, heart rate variability (HRV), derived by fast Fourier transformation, is reported as the ratio of low-frequency (LF, 0.195–0.605 Hz) to high-frequency (HF, 0.605–2.5 Hz) bands (27). This index correlates with catecholamine measurements (28), and permits the continuous assessment of autonomic balance in conscious animals (29). For HRV-analysis, we used the Kubios HRV-software (version 2.1, Biosignal Analysis and Medical Imaging Group, Department of Applied Physics, University of Eastern Finland, Kuopio, Finland) (30).

### Activity measurement

Acute LV failure was assessed by voluntary motor activity, recorded by the analysis program (A.R.T. 2.2, *DSI*). The software records strength-variations in the telemetry-signal, in relation to the location of the animal; changes in signal-amplitude are depicted as counts, the number of which depends on total animal activity. This variable correlates with the functional status, and has been used as a surrogate marker for heart failure (27, 31, 32).

### Statistical analysis

Values are presented as mean ± SEM. Kaplan–Meier survival curves were constructed and differences in mortality were assessed with Gehan’s Wilcoxon test. Categorical variables were compared with Fisher’s exact (two-tailed) test. Continuous variables were compared with analysis of variance, whereas their changes over time were compared with analysis of variance for repeated measures, both followed by *post hoc* Newman–Keuls multi-stage test. Statistical significance was defined at an alpha level of 0.05.

## Results

### Mortality

**Figure 1:**
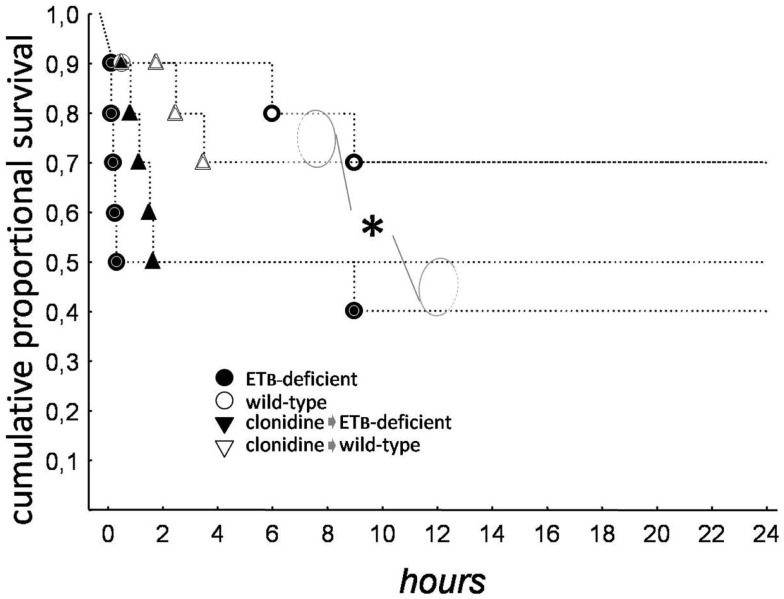
**Kaplan–Meier survival curves**. Total mortality was higher in ETB-deficient (asterisk), than in wild-type rats.

As seen in Kaplan–Meier survival curves (Figure 1), mortality was higher (*p* = 0.028) in ETB-deficient than in wild-type rats. Clonidine had no overall effect, but a shift in the mode of death was observed, from tachyarrhythmic (83% of mortality in ETB-deficient and 33% in wild-type rats) to bradyarrhythmic (100% of mortality in both groups); this difference reached statistical significance (*p* = 0.0152) in ETB-deficient rats.

### Sympathetic activation

**Figure 2:**
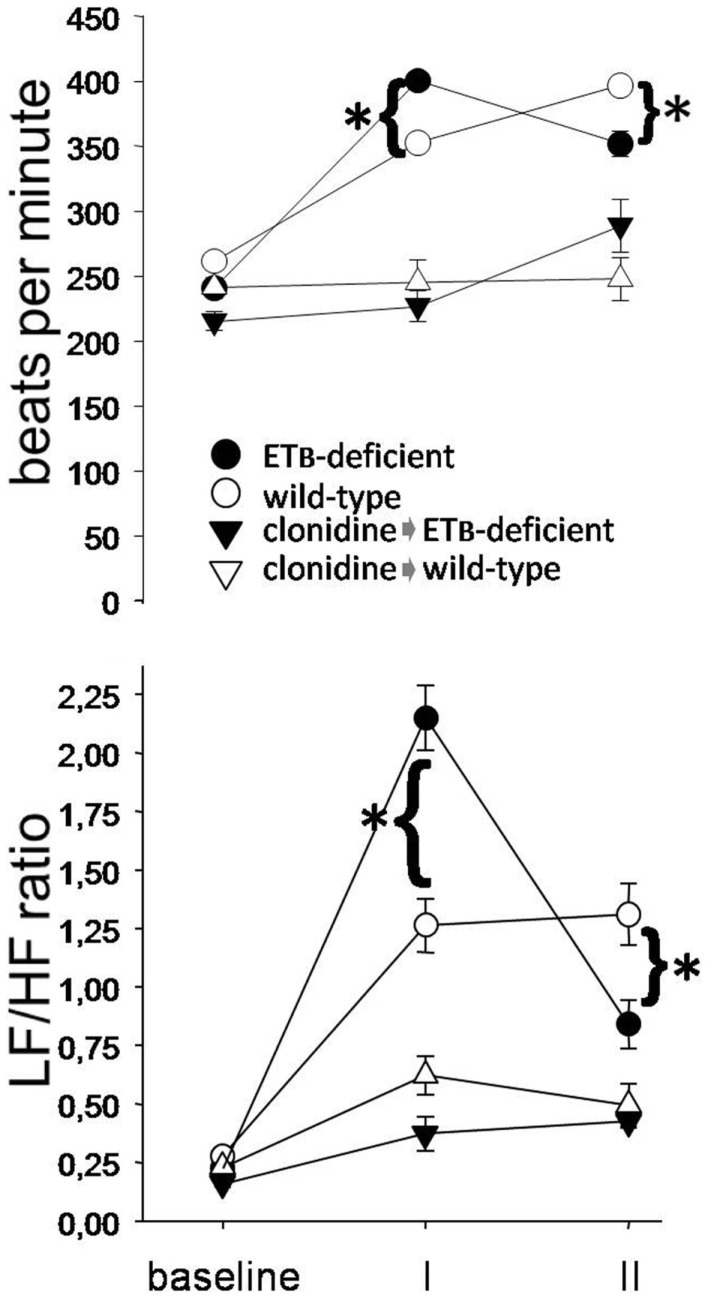
**Sympathetic activation**. Heart rate (upper panel) and low (LF) to high (HF) frequency ratio (lower panel) were higher in ETB-deficient rats during phase I (asterisks). Note their marked decrease after clonidine.

During *phase I*, HR and LF/HF ratio were higher in ETB-deficient than in wild-type rats (Figure 2). Compared to the respective untreated groups, both variables were lower after clonidine at baseline and during phase I, without differences between groups. During *phase II*, HR and LF/HF ratio were lower in ETB-deficient than in wild-type rats. After clonidine, LF/HF ratio was similar, although HR tended to be higher (*p* = 0.064) in ETB-deficient rats.

### Ventricular arrhythmias and A–V block

**Figure 3:**
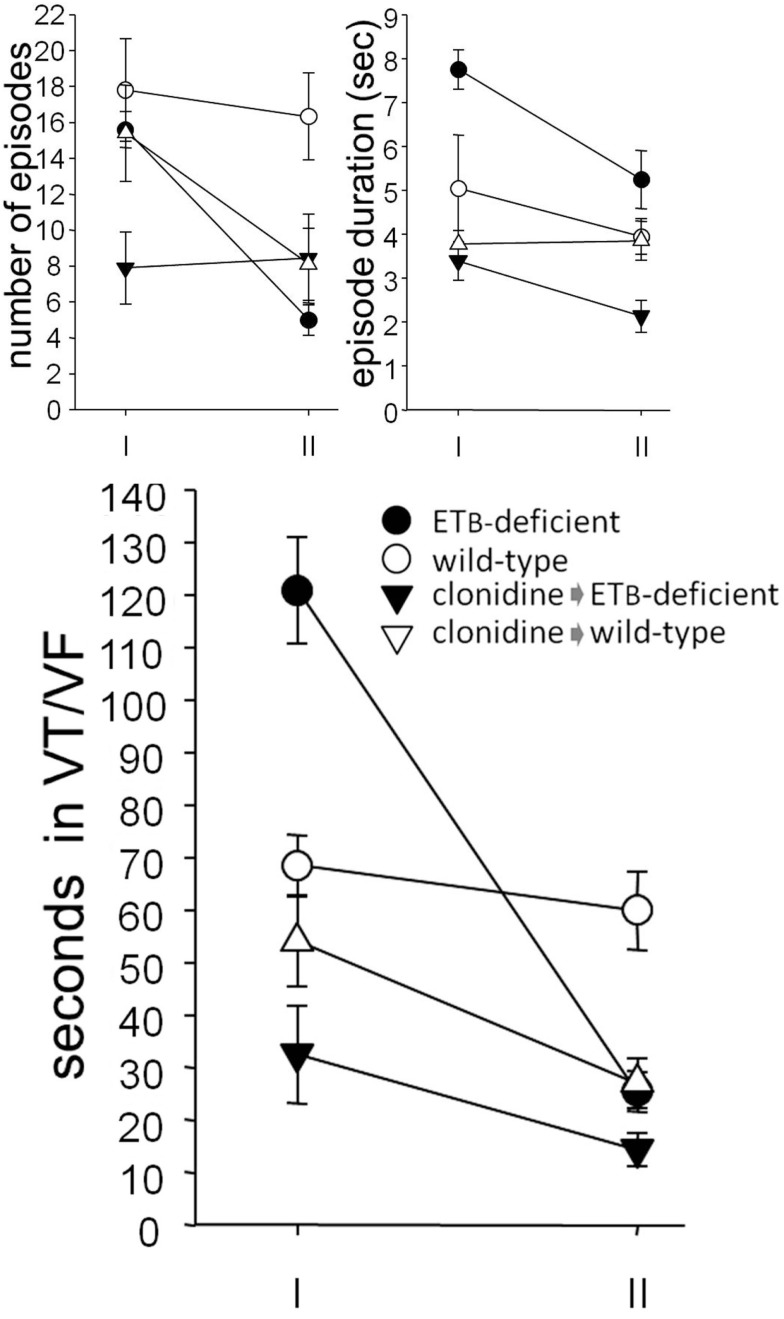
**Ventricular arrhythmogenesis**. Number and mean duration of ventricular tachycardia (VT) and fibrillation (VF) episodes (upper panel). Note, the decreased total duration (lower panel) in clonidine-treated ETB-deficient rats during phase I, and in clonidine-treated wild-type rats during phase II.

During *phase I*, the number of VT/VF episodes was comparable in the two groups, but their mean duration was longer in ETB-deficient rats, resulting in longer (*p* = 0.00013) total VT/VF duration. Compared to the respective untreated group, VT/VF duration did not change significantly in clonidine-treated wild-type rats, although variable responses were observed. In contrast, VT/VF duration decreased in ETB-deficient rats (*p* = 0.000159) after clonidine, resulting from (marginally) fewer (*p* = 0.053) and (mainly) shorter (*p* = 0.000641) episodes (Figure 3). Of note, transient second degree atrioventricular block was seen after clonidine in both groups, namely, 1.0 ± 1.0 (of 8.7 ± 8.7 s duration) and 3.7 ± 2.4 (18.5 ± 13.5 s) episodes in ETB-deficient and wild-type rats, respectively; these differences (between groups) did not reach statistical significance.

Compared to wild-type rats, less (*p* = 0.0031) VT/VF episodes occurred in ETB-deficient rats during *phase II*, resulting in shorter (*p* = 0.0010) total duration. After clonidine pretreatment, VT/VF duration was comparable in the two groups, due to decreased values (*p* = 0.0014) in wild-type rats. As in phase I, a mean of 0.7 ± 0.4 (of 1.8 ± 1.1 s duration) and 2.4 ± 1.3 episodes (10.5 ± 9.0 s) of second degree atrioventricular block were seen in ETB-deficient and wild-type rats, respectively; again, these differences (between groups) did not reach statistical significance.

**Figure 4:**
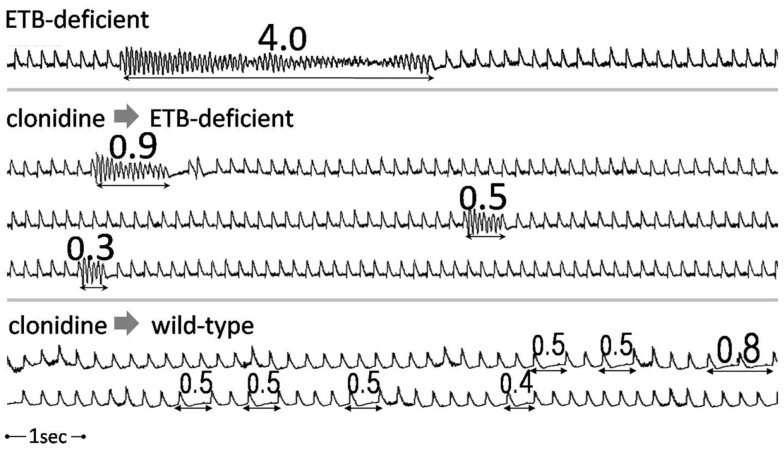
**Examples of tachy- and bradyarrhythmias**. Examples of ventricular arrhythmias (continuous strips) from an untreated (upper panel) and a clonidine-treated (middle panel) ETB-deficient rat. Frequent episodes of transient atrioventricular conduction abnormalities in a clonidine-treated wild-type rat (lower panel). Numbers indicate duration in seconds.

When normalized for survival, total VT/VF duration (during both phases) was markedly higher in untreated ETB-deficient rats (414.6 ± 162.8 s), compared to the remaining three groups [i.e., untreated wild-type rats (26.1 ± 18.9 s, *p* = 0.0020), clonidine-treated ETB-deficient rats (6.3 ± 2.7 s, *p* = 0.0063) and clonidine-treated wild-type rats (7.8 ± 2.5 s, *p* = 0.0035)]. Representative examples of VT/VF and atrioventricular block are shown in Figure 4.

### Activity

**Figure 5:**
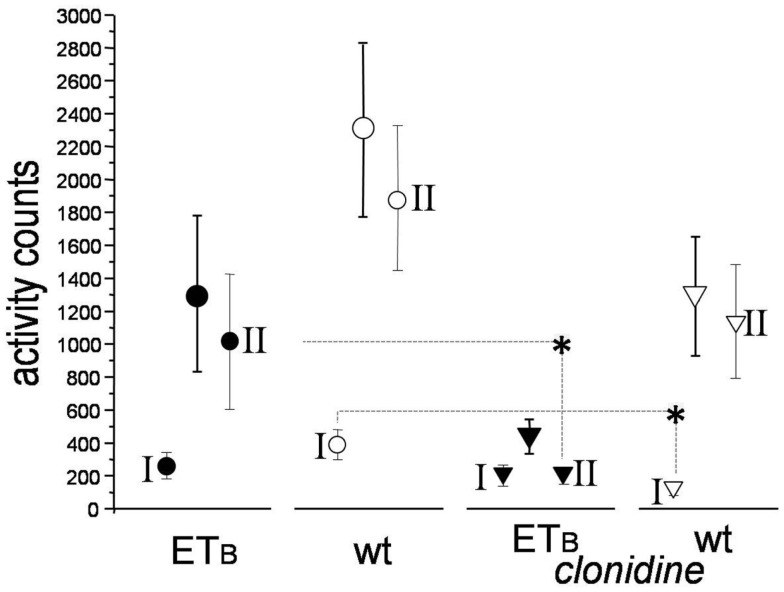
**Activity-counts**. Compared to the respective untreated groups, activity-counts were lower in clonidine-treated wild-type rats during phase I and in clonidine-treated ETB-deficient rats during phase II (asterisks).

Activity-counts were lower in ETB-deficient (1307 ± 472) than in wild-type rats (2296 ± 524) during the entire observation period post-MI, albeit of marginal statistical significance (*p* = 0.088, Figure 5). Clonidine did not change activity-counts in ETB-deficient rats during phase I, but decreased them (*p* = 0.036) in wild-type rats. Compared to the respective untreated groups, lower values were found after clonidine in both groups during phase II, but differences reached statistical significance (*p* = 0.0054) only in ETB-deficient rats.

## Discussion

Sympathetic activation is an essential mechanism responsible for arrhythmogenesis during acute-MI, but the contribution of central autonomic inputs relative to local norepinephrine release remains under investigation. We examined the effects of (the centrally acting sympatholytic agent) clonidine on rhythm disturbances in the *in vivo* MI-rat-model, focusing on the modulating actions of myocardial ETB-receptors.

### Bradycardic effects of clonidine

Clonidine-pretreatment decreased HR and deteriorated atrioventricular conduction in both rat-groups throughout the observation period. Our findings are similar to those seen after intrathecal clonidine (from the spinal cord) in a canine-model of ischemia (superimposed on healed-MI and heart failure); this study (33) reported 32% reduction in HR, 22% increase in PQ interval, and 122% increase in Wenckebach cycle-length. The bradycardic effects of clonidine on the sinus and atrioventricular nodes are thought to be secondary to autonomic modulation, rather than a direct effect on cardiac tissues (33).

### Central sympathetic activation and arrhythmogenesis in wild-type rats

The effects of clonidine in wild-type rats differed between the two (post-ligation) phases. Phase I was characterized by variable responses, but clonidine had no overall effect on VT/VF duration at this stage. Thus, the involvement of central sympathetic activation on early-phase arrhythmogenesis post-MI seems to be minor. Conversely, clonidine-pretreatment decreased (by ~50%) the number and total duration of VT/VF episodes during phase II.

Using neurographic recordings from postganglionic cardiac efferent nerves in conscious dogs, Zhou et al. (14) found augmented nerve activity immediately preceding most VT/VF episodes, underscoring its role in triggering arrhythmic events. Additional arrhythmogenic action appears to be exerted via enhanced dispersion of refractoriness, demonstrated by ventricular electrograms following left stellate ganglion stimulation in anesthetized dogs, although marked variation in the individual responses was present (34, 35). Further corroborating these observations, attenuated cardiac sympathetic activity and VT/VF were reported in dogs [in the aforementioned study by Issa et al. (33)] after intrathecal clonidine. Nonetheless, the temporal pattern of arrhythmogenesis, caused by central sympathetic inputs, along with the course of acute-MI cannot be defined from these studies (14, 33–35).

In a single experiment in the ovine model, Jardine et al. (36) reported increased nerve activity 30 min after coronary occlusion, leading to VF. This topic was subsequently systematically addressed by the same researchers (37) in a group of 11 sheep; enhanced cardiac sympathetic nerve frequency was evident (a mean of) 1 h after coronary occlusion, but again, variable degree of activation was noted (37). Examined together, the findings of the present work in the context of previous studies (14, 33–37), indicate that (in the presence of ETB-receptors in the ventricular myocardium) cardiac sympathetic nerve activity participates only to a minor extent in the mechanisms underlying early-phase VT/VF, although variable responses occur; in contrast, central sympathetic activation contributes to delayed-phase arrhythmogenesis. In addition to autonomic responses, this temporal pattern can be attributed to differences in the substrate, created by ischemia versus evolving MI (1, 25). Furthermore, LV-afterload reduction [often observed as a delayed effect after clonidine administration (38)] may have contributed to this pattern, by decreasing wall-stress and, thereby, stretch-induced VT/VF (39).

The contribution of central sympathetic activation to delayed-phase arrhythmogenesis, indicated by our results, may appear contradictory to previous experiments in Langendorff-perfused rat-hearts, in which catecholamines failed to restore phase II VT/VF (40); further pointing toward this direction, relatively limited contribution of the adrenal medulla on arrhythmogenesis was recently shown in the adrenalectomized *in vivo* rat-MI-model (41). However, it should be pointed out that neurally released and circulating norepinephrine activate adrenergic-receptors at different myocardial areas, thereby producing dissimilar electrophysiologic milieu and arrhythmogenic actions (42). We feel that this important point needs further investigation in future studies.

### Central sympathetic activation and arrhythmogenesis in ETB-deficient rats

Compared to wild-type rats, the course of sympathetic activity and arrhythmogenesis differed in our ETB-deficient group, strongly suggesting modulating effects of ETB-receptors. As in previous work from our laboratory (28, 41), we found higher incidence of VT/VF in ETB-deficient rats during phase I, along with a corresponding increase in mortality. This outcome concurs also with observations in pharmacological studies, in which dual-(ETA and ETB)-blockade (43) mitigated the protective effects of ETA-receptor-blockade during this time-frame (44). Thus, functioning myocardial ETB-receptors ameliorate arrhythmogenesis during the early post-MI phase; decreased norepinephrine overflow, mediated by attenuated exocytotic release at this stage, has been proposed as a likely explanation (45).

Contrasting phase I, phase II was characterized by lower arrhythmogenesis in untreated ETB-deficient rats, in accordance with previous results (28). The explanation for this finding is difficult, but catecholamine-depletion, secondary to excessive sympathetic activation during phase I, has been put forward as a possible mechanism (41).

In the present work, we found lower incidence of VT/VF in clonidine-treated ETB-deficient rats during phase I; this resulted mainly from shorter mean episode duration, in line with the well-described sympathetic effects on the maintenance of VT/VF over the ischemic-border (34). Thus, in contrast to wild-type rats, central sympathetic inputs appear to exert an important role on early-phase arrhythmogenesis in ETB-deficient rats. This observation can be hardly explained solely by the absence of protective effects exerted by myocardial ETB-receptors, raising the possibility of enhanced central sympathetic activation in this rat strain. This notion is reinforced by cellular studies in the brain of these rats, demonstrating elevated endothelin-converting-enzyme and extracellular ET-1 levels at basal conditions (46).

The ET-system is known to be abundantly present in the central nervous system (47), where it regulates several processes, including brain stem function (48); thus, enhanced central sympathetic activation may have contributed to arrhythmogenesis in our ETB-deficient group. This hypothesis is supported by higher HR and LF/HF ratio, observed in untreated ETB-deficient animals during phase I, as these indices reflect the autonomic balance on the sinus node, irrespective of myocardial catecholamine concentrations. Further studies are needed on this intriguing topic, focusing on the complex interaction between ET-1 and central sympathetic activation.

### LV dysfunction and ETB-receptors

We observed a trend toward lower activity-counts (used as a surrogate marker of acute LV failure) in untreated ETB-deficient rats, when compared to untreated wild-type rats. The higher incidence of heart failure, implied by our findings, is in agreement with experiments in Langendorff-perfused rat-hearts in the setting of global (10) or regional (49) ischemia, demonstrating prominent LV dysfunction, caused by genetic deficiency or pharmacological blockade of myocardial ETB-receptors. The (likely) increased incidence of acute LV failure after clonidine in our experiments (offsetting the reduction in arrhythmogenesis) adds important information on this issue; although evident in both rat-groups, this effect was more pronounced in ETB-deficient rats, as suggested by a shift toward bradyarrhythmic mortality, by markedly decreased activity-counts, and by higher HR during phase II.

The pathophysiologic link between ETB-receptors and LV dysfunction (in the setting of myocardial ischemia) is poorly understood, although increased metabolic demand caused by excessive sympathetic activation has been proposed (10). Nonetheless, the (likely) higher incidence of heart failure after central sympatholytic action (in our *in vivo* experiments) point toward additional mechanisms that may be operative. Pharmacological interaction between clonidine and cardiovascular ETB-receptors is also possible, based on previous reports in rats (38), demonstrating augmented sympatholytic effects of clonidine after ETB-receptor-blockade (and the reverse after ETB-receptor-stimulation). This topic requires thorough investigation in future work, using more specific assessment of LV function and heart failure, including methods of highlighting potential underlying mechanisms.

### Strengths and limitations

The topic examined in the present work is of high clinical significance, fueled by accumulated evidence linking central sympathetic activation with acute-MI and SCD (50). We investigated the modulating effects of ET-1 on this process, driven by experimental data (7–10) and by clinical findings, demonstrating a twofold increase in plasma ET-1 levels in patients with acute coronary syndromes triggered by emotional stress (51). Focusing on the role of ETB-receptors, we used a “subtraction” rat-model that circumvents certain caveats associated with pharmacological ET-receptor-blockade (8, 45). Lastly, our data, derived from conscious animals, were not confounded by the effects of anesthesia on sympathetic activation or arrhythmogenesis. Despite these merits, four limitations should be acknowledged: *first*, implantable transmitters, utilized in our experiments, enable thorough evaluation in conscious animals, but HRV-indices reflect the net autonomic balance on the sinus node and cannot assess sympathetic and vagal discharges separately. *Second*, we assessed the incidence of heart failure based on bradyarrhythmic death rates, HR, and activity-counts. However, (particularly the latter two of) these end-points are non-specific and should be viewed only as suggestive of acute heart failure, as they may have been confounded by known actions of clonidine, despite the fact that these were likely exerted equally to both animal-groups. Specifically, increased HR may have ensued from reflex tachycardia secondary to hypotension, and low activity counts may be attributed to the sedative effects of clonidine (52). Thus, more solid evidence of depressed LV function should be sought in future studies. *Third*, we did not explore the electrophysiological mechanisms underlying VT/VF, triggered or sustained by central autonomic inputs. *Lastly*, as the pretreatment-protocol was tailored for pathophysiological investigation, implications on treatment cannot be immediately drawn.

## Conclusion

Myocardial ETB-receptors modulate autonomic responses during acute-MI. Their genetic deficiency potentiates the arrhythmogenic effects of central sympathetic activation, leading to increased arrhythmic-mortality. Clonidine induces potent sympatholytic effects and lowers the incidence of VT/VF, but possibly aggravates heart failure, particularly in the absence of ETB-receptors. Further research is needed on the central and myocardial interaction between ET-1 and sympathetic activation in the setting of ischemia/infarction.

## Conflict of Interest Statement

The authors declare that the research was conducted in the absence of any commercial or financial relationships that could be construed as a potential conflict of interest.
